# Treatment outcomes for oropharyngeal cancer: findings from an institutional study

**DOI:** 10.3389/fonc.2026.1720966

**Published:** 2026-02-16

**Authors:** Changshu Wang, Jérôme Costisella, Benoit Emond-Guilbert, Isabelle Gauthier, Pierre-Hugues Fortier, Sameh Geha, Ayman Jafar Oweida, Mathieu Belzile

**Affiliations:** 1 Department of Medical Imaging and Radiation Sciences, Université de Sherbrooke, Sherbrooke, Québec, QC, Canada; 2 Department of Surgery, Centre Intégré Universitaire de Santé et de Services Sociaux du Saguenay-Lac-St-Jean, Saguenay, Québec, QC, Canada; 3 Department of Surgery, Université de Sherbrooke, Sherbrooke, Québec, QC, Canada; 4 Department of Pathology, Université de Sherbrooke, Sherbrooke, Québec, QC, Canada

**Keywords:** head and neck squamous cell carcinoma, human papillomavirus (HPV), oropharyngeal cancer, radiochemotherapy, radiotherapy

## Abstract

**Introduction:**

Locally advanced oropharyngeal squamous cell carcinoma (OPSCC) represents a growing clinical challenge, primarily due to the increasing incidence of human papillomavirus (HPV)-associated disease. While treatment options include radiotherapy (RT) alone, RT with Cetuximab (RCX), or radiochemotherapy (RCT), the latter remains the standard of care. This retrospective study analyzed 359 patients with locally advanced OPSCC treated at CIUSSS de l’Estrie–CHUS since 2011 to evaluate how HPV status influences outcomes across different treatment regimens.

**Methods:**

Patients diagnosed between August 2011 and September 2022 with histologically confirmed OPSCC, known p16 status, and treated with curative intent were included. Treatment modalities consisted of RT alone, RCX, RCT with weekly or 3-weekly cisplatin, or RT combined with immunotherapy. Survival outcomes were compared based on HPV status and treatment type.

**Results:**

Of the 359 eligible patients, 86.4% were HPV-positive. Five-year overall survival (OS) and disease-free survival (DFS) were markedly higher in HPV-positive versus HPV-negative patients (OS: 80.4% vs. 35.4%, p<0.0001; DFS: 76.7% vs. 28.8%, p<0.0001). Among HPV-positive patients, RCT achieved superior outcomes compared to RT alone or RCX (5-year OS: 86.6% vs. 67.7% and 73.0%, p=0.0007; DFS: 83.0% vs. 62.1% and 63.8%, p=0.0011). Within RCT, 3-weekly cisplatin yielded better OS (92.6%) than weekly cisplatin (77.4%). In HPV-positive AJCC 8th edition N1 patients, those previously staged as N2b under AJCC 7th showed a trend toward inferior survival (74.5% vs. 86.2%, p=0.057). Multivariable analysis confirmed HPV positivity as an independent favorable prognostic factor (OS HR = 0.31, 95% CI 0.19–0.51, p<0.0001; DFS HR = 0.33, 95% CI 0.21–0.52, p<0.0001). RCT significantly improved OS (HR = 0.55, 95% CI 0.33–0.92, p=0.024), while heavy smoking was associated with worse DFS (HR = 1.9, 95% CI 1.1–3.27, p=0.022).

**Conclusion:**

This study demonstrates excellent outcomes for HPV-positive OPSCC, consistent with RTOG 0129 and 1016 trials. RCT, particularly with 3-weekly cisplatin, was associated with superior survival outcomes, with an absolute 15% difference in 5-year OS in HPV-positive patients compared with weekly cisplatin. RCT remains the standard of care, while HPV-negative disease continues to pose a major therapeutic challenge.

## Introduction

The incidence of oropharyngeal squamous cell carcinoma (OPSCC) has increased substantially over the past two decades, largely driven by the rising prevalence of human papillomavirus (HPV)–associated disease. In Western countries, HPV-positive OPSCC now represents the majority of newly diagnosed cases, with projections suggesting a continued increase of approximately 30% by 2030 (1). This epidemiological shift has transformed OPSCC into a major public health concern, particularly affecting younger patients with fewer traditional risk factors such as tobacco and alcohol use.

The prognosis for OPSCC has significantly improved with the introduction of concomitant chemotherapy to definitive radiotherapy (RT) (2). Large randomized trials and meta-analyses have demonstrated that radiochemotherapy (RCT) provides superior locoregional control and overall survival compared to RT alone, albeit at the cost of increased acute and late toxicities (3). The combination of RT and cetuximab (RCX) demonstrated better survival compared to RT alone (4) but is now generally considered inferior to cisplatin-based RCT (5–7). However, the optimal cisplatin dosing schedule remains an area of active investigation. The debate over whether 3 weeks of cisplatin is equivalent to weekly cisplatin regimen during RT remains unresolved (8–12). Additionally, HPV status plays a crucial role in determining treatment outcomes (13), and numerous trials are exploring the potential for de-escalating treatment intensity in this patient population, including changing the chemotherapy regimen, and reducing the RT dose or volume (5–7, 14–16).

In this study, we report the outcomes of a retrospective analysis involving patients with OPSCC, who were treated with either RT alone, RCT, or RCX at our institution since 2011.

## Methods

Our cohort of patients was recruited between Aug 1, 2011 and Sept 30, 2022 at the CIUSSS de l’Estrie-CHUS. All patients were diagnosed with locally advanced OPSCC and were treated with curative intent.

Patient eligibility criteria: Patients were eligible for inclusion if they had histologically confirmed OPSCC, known p16 (HPV) status. All patients were required to have complete baseline staging, including imaging, pathology reports, and documented follow-up.

Curative treatments were: RT alone, RCX, RCT with 3-weekly cisplatin, RCT with weekly cisplatin or RT plus immunotherapy. Patients who received induction chemotherapy with either TPF (docetaxel, cisplatin, and 5-fluorouracil) or TP (docetaxel and cisplatin), followed by definitive RT with weekly carboplatin, were included.

Patients were excluded if they had missing HPV/p16 status, incomplete clinical or follow-up data, non-oropharyngeal primary tumors, or were treated with palliative intent. Patients who received robotic surgery or salvage surgery were also excluded.

The AJCC Cancer Staging version 8 was used for tumor staging (17).

Treatment toxicities were graded using version 5 of the Common Terminology Criteria for Adverse Events (CTCAE v5.0) (18).

Continuous variables are presented as mean ± standard deviation. Nominal variables are presented by frequencies (%). Survival analyses were performed using Kaplan-Meier curves and comparisons were made using log-rank tests. Multivariable analyses were completed using Cox models with 95% confidence intervals.

Results were obtained using R software (R Core Team, 2023).

This retrospective study has been approved by the REB of CIUSSS de l’Estrie-CHUS (approval # 2025-5719).

## Results

A cohort of 359 patients were selected according to the eligibility criteria of the study. Patient characteristics are shown in **Table 1**. Most patients were male (76.3%) with stage I disease (44.6%).

**Table 1:** Summary of patient characteristics.

Patient characteristic (n=359)	N (%)
Sex	
Male	274 (76.3%)
Female	85 (23.7%)
Age (years)	
Mean age	68.6
Median age	68.0
Tobacco	
Non-smoker	105 (29.2%)
<10 pack-years	53 (14.8%)
10–20 pack-years	35 (9.7%)
>20 pack-years	166 (46.2%)
HPV status	
p16 positive	310 (86.4%)
p16 negative	49 (13.6%)
Tumor site	
Tonsil	213 (59.3%)
Basal tongue	129 (35.9%)
Soft palate	8 (2.2%)
Posterior wall	4 (1.1%)
Oropharynx, not specified	5 (1.4%)
Tumor Staging	
I	160 (44.6%)
II	114 (31.8%)
III	51 (14.2%)
IV	34 (9.5%)
Treatment	
RT alone	62 (17.3%)
RCT	232 (64.6%)
RCX	60 (16.7%)
RT + immunotherapy	5 (1.4%)

We observed a high incidence of HPV infection (86.4%) and a significant population of heavy smokers (>20 pack-years: 46.2%). Over 60% of patients were treated with RCT (**Table 1**).

RCX was used principally between 2011–2015 at the discretion of the treating oncologist.

Neoadjuvant chemotherapy was administered only in 9 patients with TPF or TP. This treatment was followed by RT with weekly carboplatin. These cases were classified into the group treated with weekly carboplatin for the purpose of our analysis.

Combined RT-immunotherapy was used in only 5 patients who were enrolled in a clinical trial.

**Table 2** shows the proportion of patients treated with RT based on the type of chemotherapy received. 3-weekly cisplatin and weekly cisplatin were the principal treatment regimens (52.2% and 39.2% respectively). Combination with carboplatin and 5-FU (Calais study) was only used in rare cases in our institute.

**Table 2:** Types of chemotherapy combined with RT.

Standard 3-weekly cisplatin	Weekly cisplatin	Weekly carboplatin	Calais study (carboplatin+5-FU)
121	91	17	3
52.2%	39.2%	7.3%	1.3%

Among patients treated with RT and 3-weekly cisplatin chemotherapy (121 patients), the majority received 2 (55 pts, 45.5%) or 3 (47 pts, 38.8%) cycles. Some patients had weak tolerance and received only one cycle (19 pts, 15.7%).

**Figure 1:**
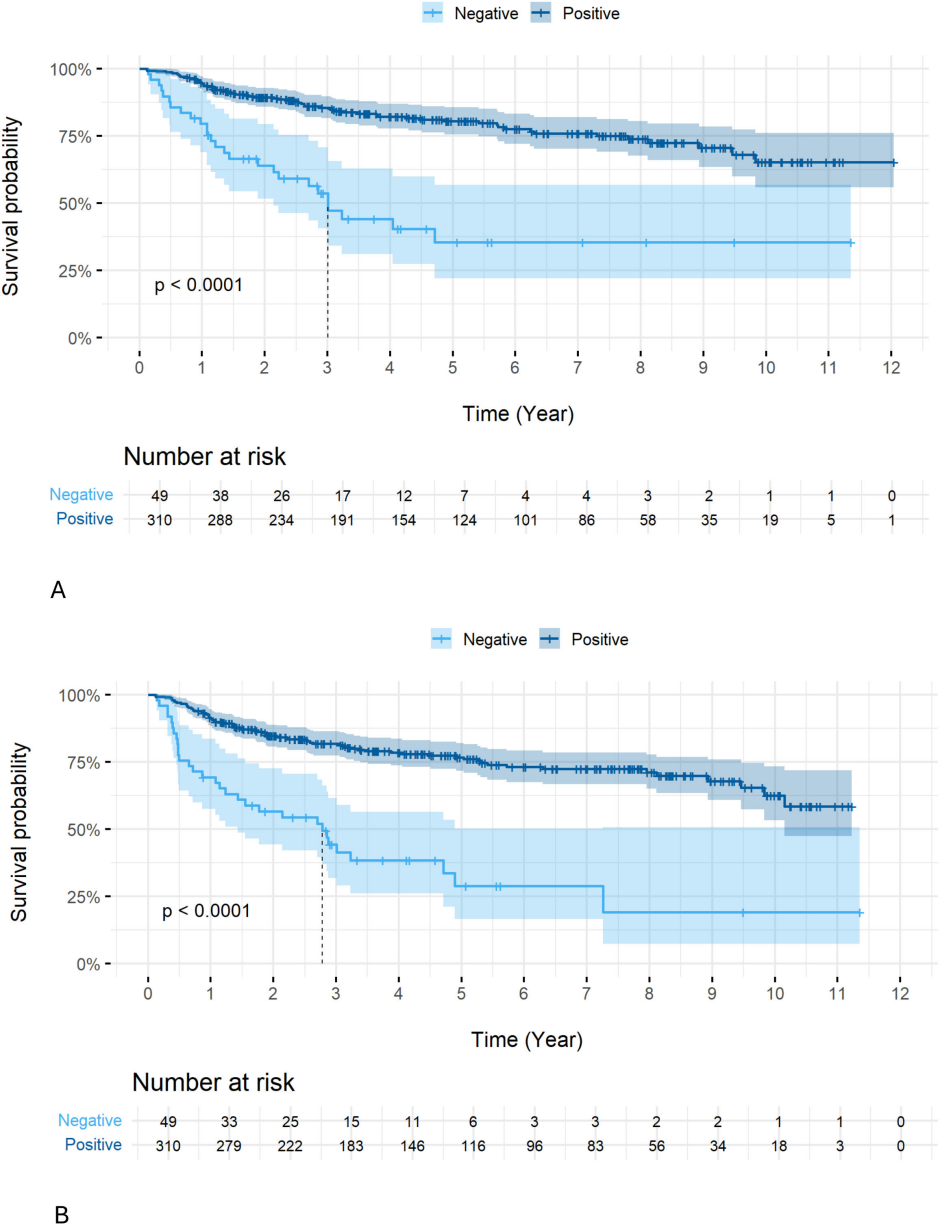
Overall OS **(A)** and DFS **(B)** according to HPV status in all patients. Overall survival (OS) was significantly better in patients with early-stage disease (stages I–II) than in those with late-stage disease (stages III–IV) across the entire cohort (5-year survival for stages I, II, III, IVA, and IVB: 95.6, 95.6, 86.1, 78.4, and 66.7, respectively; p < 0.0001). However, no significant difference in OS was observed among HPV-positive patients across stages (5-year survival for stages I, II, and III: 95.6, 95.3%, and 87.3, respectively; p = 0.67) (**Figures 2A, B**).

HPV+ patients showed a better OS (5-year OS 80.4% vs. 35.4%, p<0.0001) (**Figure 1A**) and DFS (5-year DFS 76.7% vs. 28.8%, p<0.0001) (**Figure 1B**) compared to HPV-negative patients.

**Figure 2:**
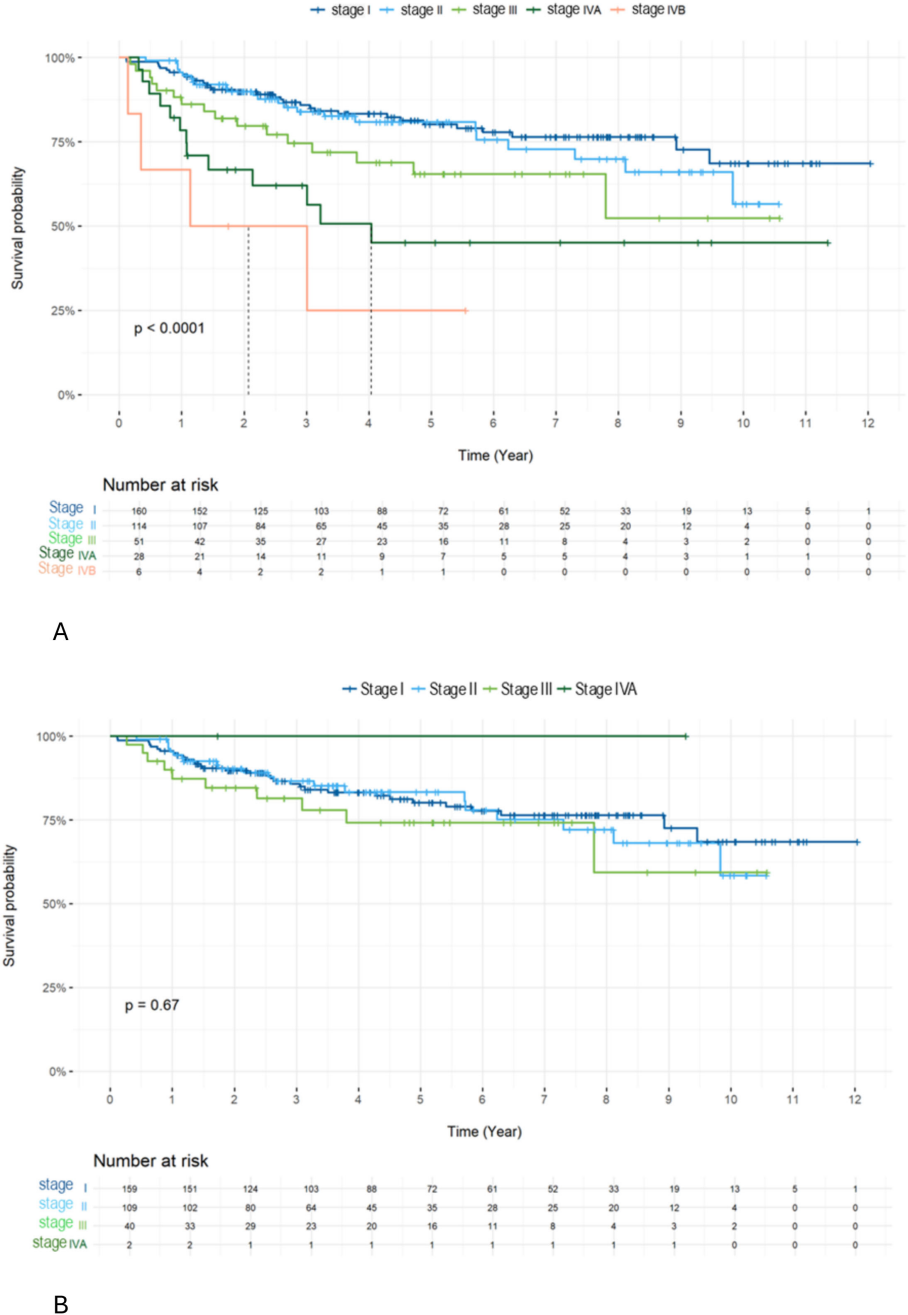
OS in all patients **(A)** and in HPV+ patients **(B)** across stages. Comparing treatment regimens, OS (**Figure 3A**) and DFS (**Figure 3B**) were better in the group treated with RCT compared to other groups. The 5-year OS was 78.8 in the RCT group compared to 66.5 and 69.2 (p=0.016) for the RCX and RT alone groups, respectively. The 5-year DFS was 74.7 in the RCT group compared to 59.8 and 64.0 (p=0.021) in the RCX and RT alone groups, respectively.

Overall survival (OS) was significantly better in patients with early-stage disease (stages I–II) than in those with late-stage disease (stages III–IV) across the entire cohort (5-year survival for stages I, II, III, IVA, and IVB: 95.6%, 95.6%, 86.1%, 78.4%, and 66.7%, respectively; p < 0.0001). However, no significant difference in OS was observed among HPV-positive patients across stages (5-year survival for stages I, II, and III: 95.6%, 95.3%, and 87.3%, respectively; p = 0.67) (**Figures 2A, B**).

**Figure 3:**
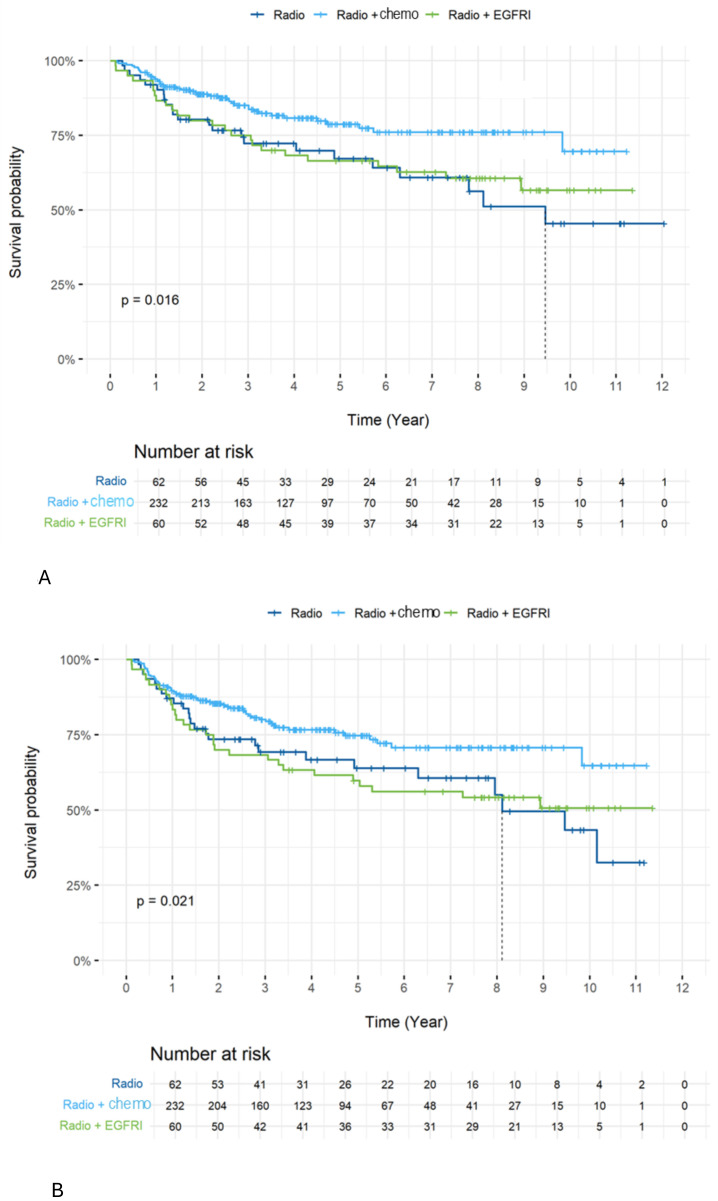
Overall OS **(A)** and DFS **(B)** according to treatment type (RCT vs RCX vs RT alone). We further investigated the impact of the type of chemotherapy received on OS and DFS. Patients who received weekly cisplatin were older, had greater hearing impairment and a lower glomerular filtration rate (GFR) (**Table 3**). We did not observe a difference in OS (**Figure 4A**) (5-year OS 86.9 vs. 72.3 p=0.057) or DFS (**Figure 4B**) (5-year DFS 80.8 vs. 70.3% p=0.13) when comparing the various chemotherapy treatment regimens. Only a tendency towards significance in OS was observed in favor of the RT with 3-weekly cisplatin chemotherapy group.

**Table 3:** Characteristics of patients treated with cisplatin chemotherapy (3-weekly vs weekly) combined with RT.

	Standard cisplatin (3-weekly)	Weekly cisplatin	P-value
	121 (57.1%)	91 (42.9%)	
Mean age Median age	63.9 64 (59.7-68)	69.2 69.7 (62.9-76)	<0.0001
Stage I	42 (34.7%)	43 (47.3%)	0.415
Stage II	45 (37.2%)	29 (31.9%)	
Stage III	21 (17.4%)	11 (12.9%)	
Stage IV	13 (10.7%)	8 (8.8%)	
HPV+	105 (86.8%)	80 (88%)	0.838
HPV-	16 (13.2%)	11 (12.1%)	
ECOG			
0 1 2 unspecified	85 25 2 9	59 21 0 11	0.415
Hearing impairment			
Normal 0–20 dB	10	5	
Mild 21-40dB	44	20	
Moderate 41-70dB	53	34	0.002
Severe 71-90dB	10	23	
Profound >90 dB	4	8	
GFR (mean ± SD)	94.53 ± 2.08	87.45 ± 3.122	0.00013

**Figure 4:**
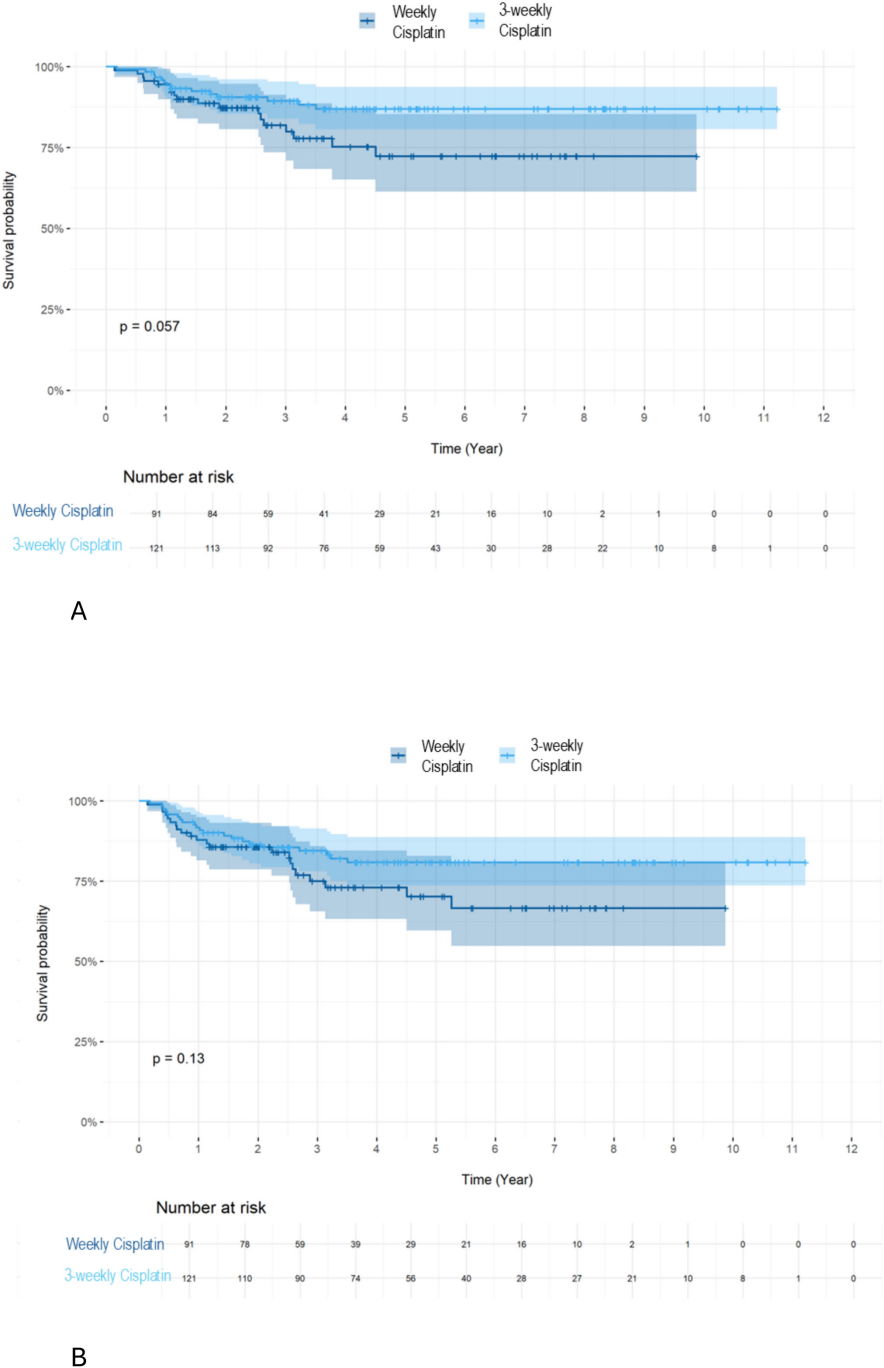
Survival rate: OS **(A)** and DFS **(B)** by 3-weekly or weekly cisplatin combined with RT in all patients. We further investigated the influence of chemotherapy type on OS based on HPV status. For HPV+ patients, we observed a superior OS (**Figure 5A**, 5-year OS 92.6 vs. 77.4 p=0.035) but not DFS (**Figure 5B**, 5-year DFS 87.3 vs. 75.7% p=0.099) using the 3-weekly cisplatin combined with RT compared to weekly cisplatin combined with RT.

**Figure 5:**
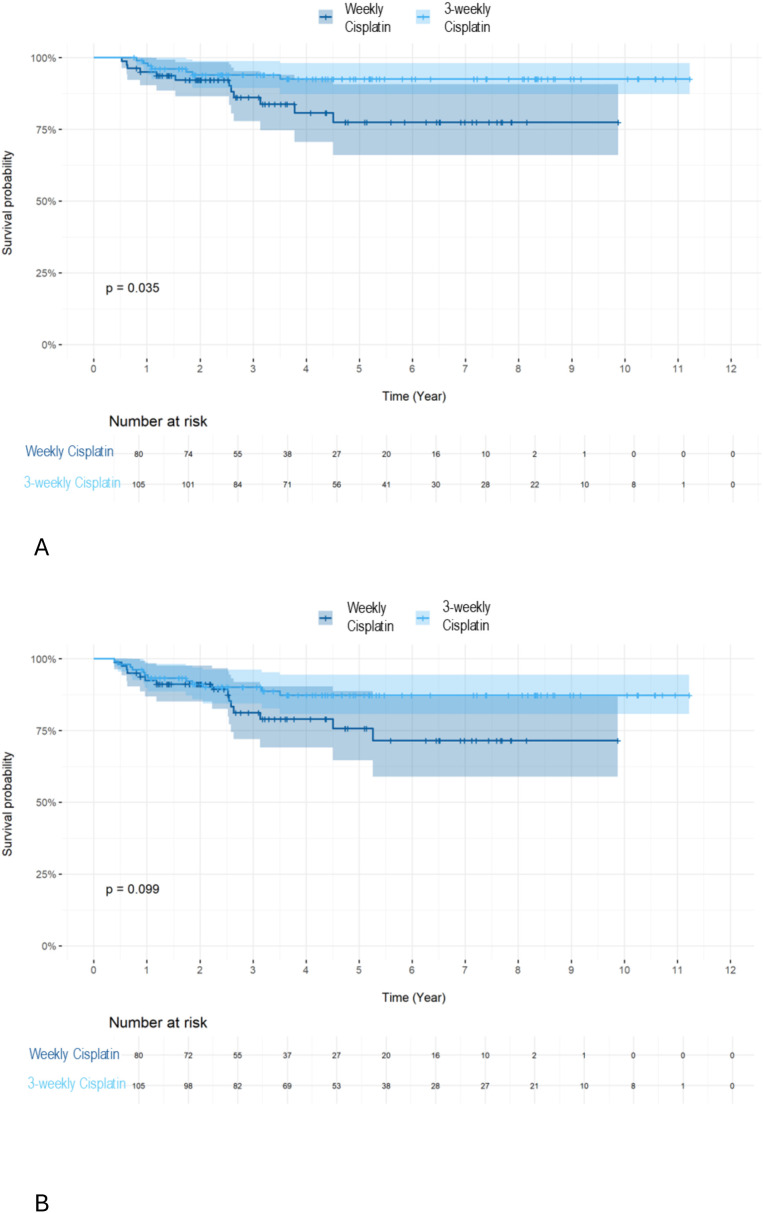
Survival rate: OS **(A)** and DFS **(B)** of 3-weekly or weekly cisplatin combined with RT in HPV+ patients. For HPV-negative patients, no difference in OS (5-year OS 46.8 vs. 33.2 p=0.57) or DFS (5-year DFS 38.6 vs. 30.3 p=0.49) was observed when comparing the two cisplatin regimens.

Comparing treatment regimens, OS (**Figure 3A**) and DFS (**Figure 3B**) were better in the group treated with RCT compared to other groups. The 5-year OS was 78.8% in the RCT group compared to 66.5% and 69.2% (p=0.016) for the RCX and RT alone groups, respectively. The 5-year DFS was 74.7% in the RCT group compared to 59.8% and 64.0% (p=0.021) in the RCX and RT alone groups, respectively.

We further investigated the impact of the type of chemotherapy received on OS and DFS. Patients who received weekly cisplatin were older, had greater hearing impairment and a lower glomerular filtration rate (GFR) (**Table 3**). We did not observe a difference in OS (**Figure 4A**) (5-year OS 86.9% vs. 72.3% p=0.057) or DFS (**Figure 4B**) (5-year DFS 80.8% vs. 70.3% p=0.13) when comparing the various chemotherapy treatment regimens. Only a tendency towards significance in OS was observed in favor of the RT with 3-weekly cisplatin chemotherapy group.

We further investigated the influence of chemotherapy type on OS based on HPV status. For HPV+ patients, we observed a superior OS (**Figure 5A**, 5-year OS 92.6% vs. 77.4% p=0.035) but not DFS (**Figure 5B**, 5-year DFS 87.3% vs. 75.7% p=0.099) using the 3-weekly cisplatin combined with RT compared to weekly cisplatin combined with RT.

For HPV-negative patients, no difference in OS (5-year OS 46.8% vs. 33.2% p=0.57) or DFS (5-year DFS 38.6% vs. 30.3% p=0.49) was observed when comparing the two cisplatin regimens.

We also analyzed chemotherapy toxicities in our patient cohort. **Table 4** shows compliance and treatment adverse events, according to the CTCAE v5.0. Prolonged hospitalization, cycle diminution of chemotherapy and GFR reduction were observed more frequently in the patients receiving 3-weekly cisplatin (**Table 4**).

**Table 4:** Adverse events of RCT according to the type of chemotherapy.

	3-weekly cisplatin(N = 121)	Weekly cisplatin(N = 91)	P value
Hearing impairment			
No significant impairment	38	30	0.423
Grade 1	19	7	
Grade 2	2	1	
Grade 3	20	3	
unspecified	42	50	
Nadir GFR (mean+CI%)	61.12 ± 6.71	70.43 ± 6.16	0.003
Neutropenia	N=82	N=52	
Grade 1	9	9	0.605
Grade 2	34	17	
Grade 3	38	20	
Grade 4	11	6	
Febrile neutropenia hospitalized in our center	N=3	N=5	
Grade 3	2	5	
Grade 4	0	0	0.386
Grade 5	1 (death)	0	
Oral mucositis			
Grade 1	9	3	0.347
Grade 2	13	13	
Grade 3	93	72	
unspecified	6	4	
Hospitalization days (mean ± CI%)	10.11 ± 14.5	6.70 ± 2.43	0.014
Parenteral feeding			
Yes	65	52	0.385
No	56	35	
unspecified		4	
Chemotherapy delayed			
Yes	19	13	0.801
No	102	77	
unspecified	0	1	
Dose diminution of chemotherapy			
Yes	20	13	0.801
No	101	78	
Cycle diminution of chemotherapy			
Yes	47	50	0.002
No			
unspecified	74 0	32 9	

**Figure 6:**
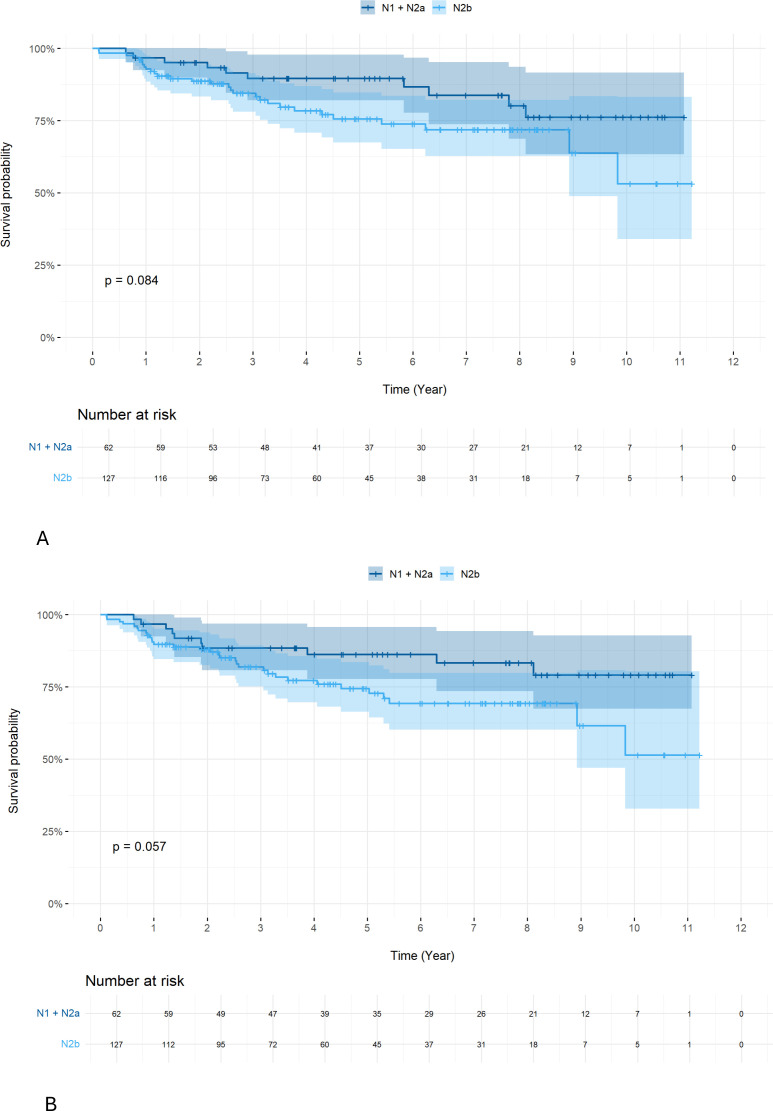
Survival rate: OS **(A)** and DFS **(B)** according to N1 status in HPV+ patients. Analysis of the impact of smoking status demonstrated that those who smoked >20 pack-years had a lower 5-year OS (**Figure 7A**; 64.2, p=0.0068) and 5-year DFS (**Figure 7B**; 58.6, p=0.0041).

**Figure 7:**
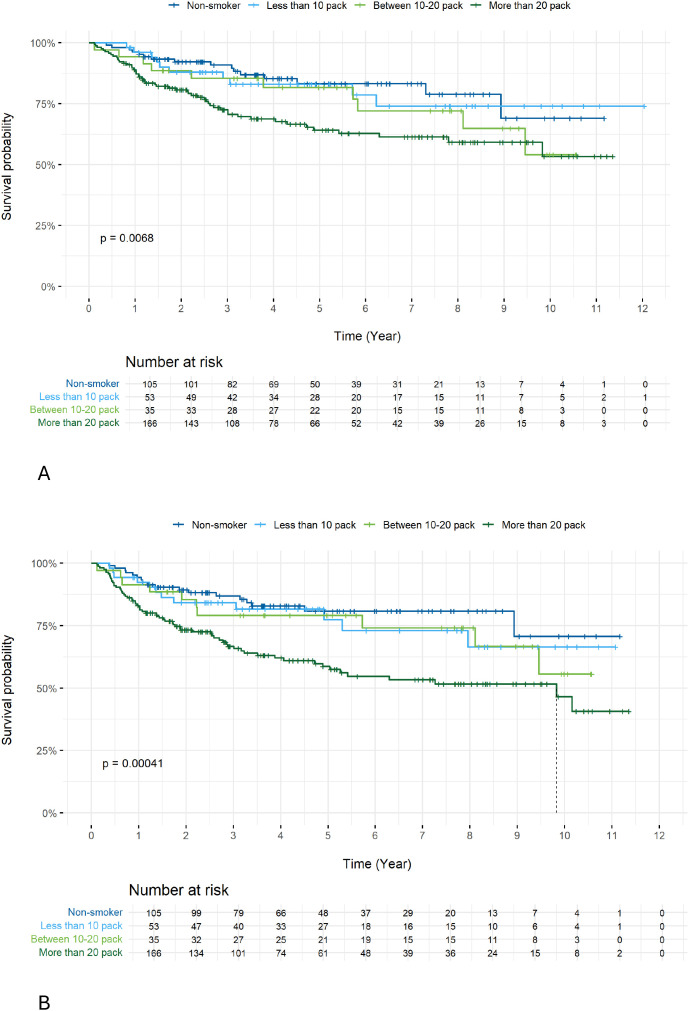
Survival rate: OS **(A)** and DFS **(B)** according to smoking history. Multivariate analysis confirmed HPV status as an independent prognostic factor. HPV positivity reduced the instantaneous risk of death by 70 (HR = 0.30 [0.18 - 0.51]; p <.0001. Compared to RT alone, RCT reduced the instantaneous risk of observing death by 44 (HR = 0.55 [0.33 - 0.92]; p = 0.03). While heavy smokers (> 20 pack-years) increased the instantaneous risk of observing events by 84 (HR = 1.9 [1.1 - 3.27]; p = 0.022).

We further investigated treatment outcomes by different N1 status of the AJCC version 8 in HPV+ patients. We used the previous version 7 to compare subgroups of N1+N2a vs. N2b. No difference in OS (**Figure 6A**, 5-year OS 89.6% vs. 75.6% p=0.084) but a trend towards an inferior DFS for N2b (version 7) compared to N1+N2a patients (version 7) was observed (**Figure 6B**), (5-year DFS 86.2% vs. 74.5% p=0.057). Analysis of the impact of smoking status demonstrated that those who smoked >20 pack-years had a lower 5-year OS (**Figure 7A**; 64.2%, p=0.0068) and 5-year DFS (**Figure 7B**; 58.6%, p=0.0041).

Multivariate analysis confirmed HPV status as an independent prognostic factor. HPV positivity reduced the instantaneous risk of death by 70% (HR = 0.30 [0.18 - 0.51]; p <.0001. Compared to RT alone, RCT reduced the instantaneous risk of observing death by 44% (HR = 0.55 [0.33 - 0.92]; p = 0.03). While heavy smokers (> 20 pack-years) increased the instantaneous risk of observing events by 84% (HR = 1.9 [1.1 - 3.27]; p = 0.022).

## Discussion

The management of locally advanced OPSCC has advanced considerably over the past decades, particularly with the widespread adoption of RCT. At our institution, all major treatment strategies have been implemented in accordance with international recommendations, providing an opportunity to review and compare real-world outcomes of OPSCC patients treated over a long period.

In this retrospective cohort, we observed a high prevalence of HPV+ OPSCC. This represents an important challenge for clinical oncology practice, particularly from a prevention perspective. In line with previously published data, smoking remained an adverse prognostic factor, with heavy smokers (>20 pack-years) experiencing significantly lower OS and DFS.

The rising incidence of HPV-associated OPSCC underscores the importance of primary prevention strategies. Prophylactic HPV vaccination has been shown to markedly reduce the prevalence of oncogenic HPV infections and is expected to significantly decrease the incidence of HPV-related oropharyngeal cancers over time. In Canada, publicly funded HPV vaccination programs are widely available and target both females and males, yet vaccine uptake remains suboptimal in certain populations. Continued efforts to improve vaccination coverage, public awareness, and equitable access are essential complements to therapeutic advances and may ultimately reduce the burden of HPV-associated OPSCC.

Consistent with the literature, we observed excellent outcomes in HPV-positive patients, with significantly superior OS and DFS compared with HPV-negative patients, confirming HPV status as an independent prognostic factor in OPSCC (13).

With respect to treatment modality, RCT was associated with improved OS and DFS compared with RT alone or RCX. Despite the favorable prognosis of HPV-positive disease, our findings support maintaining cisplatin-based RCT as the standard of care, in agreement with multiple randomized trials and meta-analyses (5–7).

We also compared two commonly used cisplatin schedules: 3-weekly versus weekly administration. While some recent studies have suggested that weekly cisplatin may reduce toxicity and improve treatment compliance, our data showed no statistically significant difference in OS or DFS when considering the entire cohort (10, 11). However, a trend for improved OS by RT with 3-weekly cisplatin was observed. Weekly cisplatin was preferentially selected for older patients and those with baseline hearing impairment or reduced GFR. In the group with 3-weekly cisplatin, we observed a lower nadir GFR, longer hospitalization and a reduction of chemotherapy cycles administered. The prolonged hospitalization was partly attributed to the need for pre-chemotherapy hydration and post-infusion monitoring before patients can be safely discharged. The reduced number of cycles reflects the lower tolerance to 3-weekly cisplatin with patients more frequently experiencing renal complications and a decline in overall clinical status. There is no statistical difference in febrile neutropenia, but these numbers only include cases in our hospital and can miss hospitalizations elsewhere. Overall, the observed 15% improvement in OS with 3-weekly cisplatin supports its continued use in HPV-positive patients when clinically feasible and argues against treatment de-intensification outside of clinical trials.

Although patients with different AJCC stages were included in the survival analyses, this approach reflects real-world clinical practice in OPSCC, where treatment selection is primarily driven by tumor site, HPV status, nodal burden, and patient-related factors rather than stage alone. In particular, HPV-positive OPSCC demonstrates favorable outcomes across multiple stages, and excessive stratification by individual AJCC stages in retrospective cohorts may result in underpowered and unstable estimates. To address this limitation, we performed focused subgroup analyses based on nodal burden and HPV status, which are clinically relevant prognostic determinants. Since the 8th edition of the AJCC cancer staging manual, all ipsilateral lymph nodes smaller than 6 cm are classified as N1, encompassing N1, N2a, and N2b categories from the 7th edition, irrespective of nodal size or number. However, clinical experience from the AJCC 7th edition suggests that patients with multiple or larger lymph nodes may have less favorable outcomes despite being classified as N1 under the current system. Accordingly, we analyzed treatment outcomes in HPV-positive N1 patients (AJCC 8th edition) by further stratifying them according to AJCC 7th edition nodal subgroups (N1 + N2a vs. N2b). This analysis revealed a trend toward inferior DFS in patients previously classified as N2b (p = 0.057). These findings highlight the prognostic importance of nodal burden within HPV-positive disease and support the continued use of concurrent chemotherapy in patients with higher nodal involvement.

To further evaluate the prognostic impact of HPV status, we analyzed survival outcomes according to HPV status within the major treatment modalities. Across treatment groups, HPV-positive patients consistently demonstrated superior OS and DFS compared with HPV-negative patients, with the greatest benefit observed in patients treated with concurrent RCT. While stage-specific comparisons of HPV-positive and HPV-negative disease may be of interest, such analyses were limited by subgroup sizes in this retrospective cohort. We therefore prioritized treatment- and nodal burden–based analyses, which provide clinically meaningful insight while maintaining adequate statistical power.

Due to the small number of patients treated with RT and immunotherapy in our center, we could not analyze treatment outcomes for these patients. Results from ongoing randomized trials comparing RT plus chemotherapy with RT plus immunotherapy (KEYNOTE-412, NRG-HN005) are eagerly awaited and will help define the role of immunotherapy in the definitive treatment of head and neck squamous cell carcinoma.

Finally, treatment outcomes for HPV-negative patients remain low even with combined standard chemotherapy. This continues to pose a major therapeutic challenge, likely reflecting intrinsic radio-chemotherapy resistance and an immunologically cold tumor microenvironment. A prospective translational study is currently ongoing at our center, aiming to identify potential tumor markers that could help personalize treatment strategies and improve OPSCC patient outcomes.

## Conclusion

This retrospective study supports that RCT remains a standard treatment for HPV-positive patients, and specifically, 3-weekly cisplatin RCT offers improved survival compared to RT alone or RCX. Treatment challenges persist for HPV-negative patients with a low 5-year OS. These findings should be interpreted in the context of tumor stage, nodal burden, and HPV status. Our additional subgroup analyses focusing on HPV status and nodal burden highlight clinically relevant heterogeneity within AJCC stage groupings.

## Data Availability

The raw data supporting the conclusions of this article will be made available by the authors, without undue reservation.
